# Targeting Bone Metastasis in Cancers

**DOI:** 10.3390/cancers13174490

**Published:** 2021-09-06

**Authors:** Edith Bonnelye, Patricia Juárez

**Affiliations:** 1 Department of Efficacy and Resistance to Anti-Tumor Targeted Therapies, University of Lille, CNRS, Inserm, CHU Lille, UMR9020-U1277-CANTHER-Cancer Heterogeneity Plasticity and Resistance to Therapies, F-59000 Lille, France; 2 Biomedical Innovation Deparment, Centro de Investigacion Cientifica y de Educacion Superior de Ensenada, Ensenada 22860, Mexico; pjuarez@cicese.mx

This Special Issue of *Cancers* covers different aspects of bone physiopathology in oncology that combine the microenvironment and the factors involved in bone metastasis dormancy and progression. It also reviews several clinical aspects of bone metastases including cancer stages (neuroendocrine neoplasms and CRPC for prostate cancer), personal medicine (biomarkers and micro-RNA), treatments (beta-blockers, radiotherapy, and denosumab), and side effects (extraskeletal metastases on skeletal-related events, osteonecrosis).

The two first papers that review the bone metastasis microenvironment focus on the role of immune cells. The first paper discusses macrophages, such as tumor- and metastasis-associated macrophages (TAMs and MAMs), and their polarization process. The authors then discuss T cells, especially their role in niche establishment for metastatic cancer cells and their contribution to the progression of bone lesions [1]. Immunotherapeutic strategies that target macrophages and T cells are also presented. The second paper addresses the bone marrow adipocytes in bone cancer, describing their expansion during aging and in metabolic syndromes, such as obesity, which creates “yellow” marrow. The authors consider the origins of bone marrow adipocytes (BMAds) and their endocrine roles. The authors also discuss the role of BMAds in cancer initiation and progression and how to target these adipocytes or their secreted factors [2]. The three next articles summarize the roles of several factors in the development of osteolytic lesions. The first one, by Drescher et al. [3], describes the tyrosine kinase receptor TIE2 in breast cancer (BCa) cell dormancy in vivo and in vitro, and also its resistance to the chemotherapeutic 5-Fluorouracil. They also show that BCa patients with higher TIE2 expression are associated with an increased time to metastasis and survival. Since TIE2 is a target for anti-angiogenic treatments, its inhibition may then induce dormancy wake-up and proliferation. This is followed by a review by Salamanna et al. [4] that summarizes the role of the interleukins IL-1, IL-6, IL-8, and IL-11 in BCa bone metastases as pro-metastatic factors in bone and IL-2 and IL-12 which have an anti-metastatic effect by generating a local and systemic antitumor response. They also raise the complexity of the understanding of the interleukins’ roles and the inconsistency in clinical trials. These issues raise the need for further studies. The next article, by Omokehinde and Johnson [5], presents an overview on the glycoprotein 130 (GP130) co-receptor cytokines’, including IL-6, IL-11, LIF, OSM, CNTF, and CT-1, functions in regulating a wide range of processes that affect bone remodeling, cancer pathogenesis, and metastasis through their paracrine and autocrine mechanisms.

The second series of articles mainly presents clinical data deciphering cancer stages, personal medicine treatments, and side effects. Okuma et al. [6] begin with a single-center retrospective observational study (*n* = 123) to identify the risk for developing denosumab-related osteonecrosis of the jaw (DRONJ) in stage IV solid cancer patients with bone metastases. They found that 11.4% of patients developed DRONJ, as well as finding a positive correlation between DRONJ and hormone therapy, chemotherapy, and/or molecular targeted (angiogenesis and TK inhibitors) therapy. In the next article, Argentiero et al. [7] discuss the current knowledge on skeletal metastases of unknown primary (SMUP). SMUP is a rare metastatic tumor, which is often aggressive, where the primary site of bone metastases often remains unidentified. Clinical evidence is limited, accounting for 2% of all cancers characterized by a poor response to chemotherapy. The diagnostic and therapeutic approaches to SMUP are described along with current treatments for bone target therapies.

In the context of prostate cancer, Lobo-Martins et al. [8] present a retrospective analysis of the prospective, randomized, and multicenter clinical trial of denosumab vs. zoledronic acid in patients with metastatic castration-resistant prostate cancer (mCRPC) and bone metastases. Although the bone is the most common site of metastatic disease in prostate carcinoma, many (mCRPC) patients also present metastatic disease outside the bone, with a type of metastatic spread. Visceral metastases have long been considered a negative prognostic factor for survival in mCRPC. This paper investigates the impact of metastatic compartment bone and extraskeletal metastases vs. bone-only metastases on bone biomarker levels, the time to the first on-study skeletal-related events, and symptomatic skeletal events.

Neuroendocrine system interactions with the bone are addressed by two reviews: First, Altieri et al. [9] presents a review discussing the incidence, pathogenesis, and diagnostic and therapeutic implications of neuroendocrine neoplasms (NENs) that localize to the bone, particularly gastro-entero-pancreatic and bronchopulmonary solid tumors. NENs are a heterogeneous group of tumors arising from cells with a neuroendocrine phenotype, mainly originating from the GEP and the BP tract. NENs’ presence in bone has been considered rare due to limitations caused by the lack of quantitative detection methods. However, the improvement in therapeutic options and imaging techniques, such as positron emission tomography with gallium peptides, has improved the diagnosis of bone metastases in NENs. In the next review paper, Bernadette and Elefteriou [10] discuss mechanisms supporting the use of beta-blockers to manage BCa bone metastasis. Recently, the importance of the sympathetic nerves interacting with bone cells and their influence in the establishment and progression of BCa bone metastasis have been demonstrated. Preclinical and clinical studies in BCa patients have shown that sympathetic stimulation of β-adrenergic receptors is associated with an increased vascular bone density and the adhesion of metastatic cancer cells to blood vessels associated with chronic stress. β-blockade prevented these events in mice with high endogenous sympathetic activity, which could support the use of beta-blockers as an adjuvant therapy to increase the survival of patients with bone metastases.

The last two papers discuss diagnosis, treatment, and personalized medicine in bone metastases. Marazzi et al. [11] discuss the use of diagnostic imaging to detect bone metastases in BCa, and targeted radiotherapy and local therapy to improve bone metastases in BCa. The authors summarize the current knowledge of radiotherapy’s effect on bone metastases, offering a practical guide for multidisciplinary management of patients with BCa bone metastases. 

Lastly, Wood and Brown [12] nicely summarize the past and recent findings in biomarkers as predictors of the risk of dissemination of the primary cancers to the bone, including breast, prostate, lung, and renal cell carcinoma. Thanks to the application of molecular profiling techniques, animal models, and engineered cell lines, this area continues to show an increase in applications. It has excellent potential for the early detection of bone metastases.

We hope that this Special Issue that covers different aspects of bone metastasis processes will contribute to a better overall understanding of the different aspects of the research driven by the scientific community in osteo-oncology and will initiate new research aiming to find new treatment strategies that can improve the survival and quality of life of patients. 
